# Immune Protection Against *Chlamydia trachomatis* in Females

**DOI:** 10.1155/S106474499600035X

**Published:** 1996

**Authors:** Richard P. Morrison

**Affiliations:** Department of Medicine Division of Infectious Diseases and the Department of Microbiology University of Alabama at Birmingham 1900 University Blvd., THT 229 Birmingham AL 35294-0006 USA

## Abstract

Despite significant advances in our understanding of the biology and antigenic structure of *Chlamydia trachomatis*, and the epidemiology and clinical spectrum of chlamydial disease, the magnitude of morbidity from human chlamydial infections remains an important public health concern. Control of chlamydial disease will likely depend on a multidisciplinary approach, including the development of immunoprophylactic or immunotherapeutic strategies. Reasonable progress has been made in understanding specific immune mechanisms that contribute to host immunity in experimental models of chlamydial infection. However, studies of human immunity have not been so successful. This is particularly evident in that studies to address the development and role of mucosal immune responses to urogenital chlamydial infections have not been forthcoming. The following review is a brief summary of our current knowledge of protective immunity to chlamydial urogenital infections of females. It is not meant to be exhaustive, but instead to touch upon aspects of protective immunity that have been described in both human and experimental animal models of chlamydial infection.


Infectious Diseases in Obstetrics and Gynecology 4:163–170 (1996)
© 1996 Wiley-Liss, Inc.

### Immune Protection Against *Chlamydia trachomatis* in Females

Richard P. Morrison

*Department of Medicine, Division of Infectious Diseases and the Department of Microbiology, University of Alabama at Birmingham, Birmingham, AL*

ABSTRACT

**Despite significant advances in our understanding of the biology and antigenic structure of *Chlamydia trachomatis,* and the epidemiology and clinical spectrum of chlamydial disease, the magnitude of morbidity from human chlamydial infections remains an important public health concern. Control of chlamydial disease will likely depend on a multidisciplinary approach, including the development of immunoprophylactic or immunotherapeutic strategies. Reasonable progress has been made in understanding specific immune mechanisms that contribute to host immunity in experimental models of chlamydial infection. However, studies of human immunity have not been so successful. This is particularly evident in that studies to address the development and role of mucosal immune responses to urogenital chlamydial infections have not been forthcoming. The following review is a brief summary of our current knowledge of protective immunity to chlamydial urogenital infections of females. It is not meant to be exhaustive, but instead to touch upon aspects of protective immunity that have been described in both human and experimental animal models of chlamydial infection.** © 1996 Wiley-Liss, Inc.

KEY WORDS

*C. trachomatis,* pathogen, urethritis, immunity, cervicitis, salpingitis

*Chlamydia trachomatis* is an obligate intracellular bacterial pathogen that primarily infects the mucosal epithelium of the eye and urogenital tract. Chlamydial urogenital tract infections cause a broad range of clinical syndromes ranging from asymptomatic infection to urethritis and epididymitis in men, and cervicitis and salpingitis in women. In the US alone, it is estimated that 4 million new cases of chlamydial infection occur annually. Women account for 2–3 million of those infections of which approximately 500,000 develop chronic salpingitis, with 10% becoming infertile as a result of infection. Although chlamydial infections constitute a major public health problem worldwide, our understanding of the immune responses that contribute to protective immunity are inadequately defined.

The development of protective immunity to chlamydial infection most certainly involves the interaction of both humoral and cell mediated immune (CMI) responses. Antibody, cytokines, helper T cells, and cytotoxic T cells have been implicated as effectors in host immunity to experimental chlamydial infection.[4,8,20,46,51,59,63] Their importance, or lack of, is dependent on the animal model, route of infection, infecting chlamydial strain, and a host of other factors that vary from laboratory to laboratory. The development of chlamydial-specific antibody and cellular immune responses coincide with recovery from chlamydial infection, and therefore it is difficult to assign a predominant role to either. A consensus has not been reached regarding the preeminent protective antigen(s) or immune response(s) that confer immune protection, however significant progress has been made in regard to the

**Address correspondence to Richard P. Morrison, Ph.D., Department of Medicine, Division of Infectious Diseases, University of Alabama at Birmingham, 1900 University Blvd., THT 229, Birmingham, AL 35294-0006.**

Received September 15, 1996

Accepted October 1, 1996

Article fundamental role of anti-chlamydial antibody and cellular immune responses. Definition of protective immune responses and their cognate antigens is also not well defined in human chlamydial infection, but immune protection likely involves aspects of both antibody and cell mediated responses. It is not within the scope of this manuscript to review all aspects of immunity to chlamydial infection. Instead I will focus on, and limit my discussion to, protective immunity and aspects of the systemic and local immune responses that develop following mucosal chlamydial genital tract infection of females.

### Role of Antibodies in Protective Immunity to Mucosal Chlamydial Infection

Studies of chlamydial infection in experimental animals provide evidence that infection leads to the development of protective immunity, but resistance wanes over time and reinfection is possible.[3,37,43,50] In the guinea pig models of ocular and genital tract infection, antibodies appear to play a predominant role in protective immunity. The concept that chlamydial-specific mucosal antibody may be an important mediator of immune protection is supported by a number of studies.[29,30,32,45,57] Those studies demonstrate that ocular infection or enteric vaccination of guinea pigs with viable chlamydiae, but not parenteral immunization, protects animals from reinfection. Protection is short-lived, associated with the presence of secretory IgA, and is not transferable with immune serum. Collectively those data imply an essential role for secretory antibody in protective immunity to experimental ocular chlamydial infection.

Rank et al.,[46] using the guinea pig model of chlamydial genital tract infection, demonstrate a predominant role for antibodies in the resolution of infection and protection from reinfection. The importance of antibody in the resolution of infection was demonstrated by treating guinea pigs with cyclophosphamide to suppress antibody responses, while leaving CMI responses intact. Cyclophosphamide treated animals had intact CMI responses, but did not produce antichlamydial antibodies and did not resolve chlamydial infection. Furthermore, resolution of infection correlated with the presence of mucosal anti-chlamydial IgG and IgA antibodies.[42] Resistance to re-infection in the guinea pig model is also antibody dependent. Suppression of the antibody response with cyclophosphamide, followed by infection and subsequent treatment with tetracycline to cure infection, results in animals having intact chlamydial CMI responses, but lack chlamydial specific antibody. Subsequent challenge of those antibody-deficient animals results in infection.[41] To further elucidate the protective effect of anti-chlamydial antibody, naive guinea pigs were hyperimmunized with immune serum (Ig fraction) and subsequently challenged. Although passive immunization did not confer solid protection, shedding of infectious chlamydiae was decreased. Collectively, results from the guinea pig model of chlamydial genital tract infection suggest that CMI alone is not sufficient to confer protective immunity, and provide indirect evidence that antibody plays a protective role.

The murine models of *C. trachomatis*, strain mouse pneumonitis (MoPn), genital and respiratory tract infection have also provided useful insights into the mechanisms of protective immunity. In the murine model of chlamydial pneumoniae, antibody (immune sera) provides some degree of protection when administered locally or systemically at the time of infection.[60,61] However, mice rendered antibody deficient by treatment with anti-$\mu$ antibody are not more susceptible to primary respiratory tract infection.[58] Similarly, antibody depleted mice (anti-$\mu$ treated) resolve chlamydial genital tract infection with kinetics similar to immunocompetent mice.[40] In that study, antibody depleted mice developed chlamydial-specific delayed type hypersensitivity and T cell proliferation responses, but failed to produce either serum or secretory anti-chlamydial antibody. Furthermore, antibody depleted mice were resistant to secondary infectious challenge. Therefore, unlike the guinea pig model of chlamydial infection, where antibody plays a predominant role in protective immunity, antibody appears not to be an important aspect of protective immunity in murine genital tract infection. The apparent difference in the protective role of antibody in the mouse and guinea pig models of infection is not understood, but has led to the hypothesis that CMI responses alone are sufficient to resolve murine chlamydial genital tract infection (discussed below).[34]

In humans, indirect evidence and correlative data suggest that protective immunity does develop following naturally acquired chlamydial infection.

Early trachoma vaccine trials in humans and monkeys establish that vaccination confers some degree of resistance to the homologous chlamydial strain, but heterologous challenge leads to infections that result in more severe disease.[14,15] Few studies have examined whether naturally acquired genital tract infection in humans provokes protective immunity, but some studies suggest that prior infection may confer some level of protection. For example, the prevalence rates for acquiring chlamydial genital tract infection are higher in adolescents than in older adults.[27,47,48,49] Significantly lower isolation rates for chlamydiae are also reported for men and women who had prior chlamydial infection or nongonococcal urethritis.[1,22] Collectively, those studies imply that prior infection confers some degree of protective immunity to chlamydial infection.

The immune mechanisms that confer partial immunity to infection in humans have not been identified, but experimental infections of humans and subhuman primates with *C. trachomatis* demonstrate that protection is short-lived and serovar specific.[21,56,62] Furthermore, the presence of serovar-specific antibodies in local secretions correlates with protection to reinfection.[2,33] The precise role of antibodies in protective immunity to human chlamydial infections is undefined, but data from experimental systems show that antibodies are neutralizing. Monoclonal antibodies to both contiguous and conformational epitopes located on the chlamydial major outer membrane protein (MOMP) neutralize chlamydial infectivity for cultured eukaryotic cells, passively protect mice against chlamydial "toxicity" and prevent the infection of monkey conjunctivae,[63] and thus support the premise that antibodies might be protective in vivo.

The literature is replete with serological studies of patients with urogenital chlamydial infections,[7,10,11,12,13,31,55] but few studies provide any direct correlates with protective immunity. In one study that addresses mucosal immune response in humans, Brunham, et al.[5] analyzed the serum and secretory anti-chlamydial antibody response in women with uncomplicated *C. trachomatis* cervical infection. They found that women from which the fewest number of organisms were recovered had the highest prevalence of secretory anti-chlamydial IgA antibodies. Recovery of the organism from the cervix was inversely correlated with the presence of local anti-chlamydial IgA antibodies, and not serum antibodies. Those data suggest that the secretory antibody response may play a role in immunity to chlamydial genital tract infection, but further studies are necessary to confirm and augment those initial findings.

Although chlamydiae infect, replicate, and cause immunopathological sequelae at the genital tract mucosa, few studies have investigated the role of mucosal immune responses in human infection. Our understanding of the mucosal immune system had advanced significantly in the past decade, and therefore the opportunities exist for detailed analyses of the mucosal immune response to human chlamydial genital tract infection. Such studies will advance our understanding of the role of mucosal immune responses in protection and immunopathogenesis of chlamydial disease, and may provide important information that would be useful for the design of specific control measures for chlamydial infection.

### Role of CMI Responses in Protective Immunity to Mucosal Chlamydial Infection

Marked CMI responses, such as delayed type-hypersensitivity and antigen-specific T cell proliferation, are elicited following chlamydial infection of humans and experimental animals,[6,17,18,43,45] but little is known about the effector role of those responses in protective immunity. The most detailed information regarding the characterization and identification of *Chlamydia*-specific T cell responses comes from studies using the murine model of chlamydial genital tract infection.

Genital tract infection of mice with *C. trachomatis* results in a self-limiting infection that resolves within several weeks without antibiotic therapy, thus providing a useful model for evaluating host immunity. Rank, et al.[44] used T cell deficient nude mice to demonstrate the requirement for T cell mediated responses in resolving chlamydial genital tract infection. Genital infection of nude mice results in a chronic infection that persists for >9 months, and the adoptive transfer of spleen cells enriched for T cells or B cells brings about the resolution of infection. While the nude mouse model clearly demonstrates the need for T cells in protective chlamydial immunity, the contribution of other lymphocyte populations or subpopulations of T cells has not been resolved. In contrast to the study by Rank, et al.,[44] Tuffrey, et al.,[53,54] found that T cell deficient mice resolve chlamydial genital tract infection similar to infection of immunologically intact animals, and that the transfer of immune lymphocytes did not confer additional protection. It should be pointed out, however, that significant differences exist in the experimental design and the strain of *C. trachomatis* used for infections. For the purpose of brevity, and because there exists such dramatic differences in host immunity in these two dissimilar models of infection, the remainder of this discussion will pertain primarily to *C. trachomatis* MoPn infection.

Studies addressing the relative contribution of T cell subpopulations in resolving chlamydial genital tract infection have been inconclusive.[26,38] For example, despite in vivo depletion of either $CD4^+$ or $CD8^+$ T cell subpopulations with monoclonal antibodies, mice resolve chlamydial infection with kinetics similar to that of non-treated animals.[38] Those results suggest that either both T cell populations are capable of bringing about the resolution of chlamydial infection, or that the depletion of cell populations was incomplete. If both T cell populations are capable of resolving infection alone, then perhaps a common immune mechanism such as secretion of the chlamydiae-inhibitory cytokine interferon-$\gamma$ plays a predominant role. Recently, however, the role of $CD4^+$ cells and $CD8^+$ T cells in immune protection was re-evaluated.[52] The adoptive transfer of $CD4^+$ T cells, that were obtained from mice following the resolution of primary chlamydial genital tract infection, conferred a significant level of protective immunity to immuno-competent naive animals. In contrast, the transfer of $CD8^+$ T cells obtained from the same donor mice had no effect. Furthermore, the protective $CD4^+$ T cells secreted lymphokines characteristic of both Th1 and Th2 type T cells. Therefore, those results confirm a primary protective role for $CD4^+$ T cells, but the relative contribution of Th1 vs Th2 type helper T cell responses was not clarified. In a recent study by Cain and Rank,[9] mononuclear cells isolated from genital tract tissue of infected mice and stimulated in vitro with chlamydiae, produced a Th1-like pattern of cytokines. Those results imply that Th1-type helper T cell responses coincide with the resolution of genital tract infection. Clearly, though, the precise role of $CD4^+$ and $CD8^+$ T cells in protective immunity to chlamydial genital tract infection has not been established and other approaches must be taken to address the contribution of these cell population.

As an alternative approach to defining the capacity of various T cell subpopulation to confer immunity to chlamydial genital tract infection, T cell lines and clones have been isolated and tested. The initial testing of chlamydial specific T cell lines demonstrated that lines enriched for either $CD4^+$ or $CD8^+$ T cells were capable of resolving infection, although $CD4^+$ cells were more efficient than $CD8^+$ cell lines.[39] Subsequently, both $CD4^+$ and $CD8^+$ T cell clones have been isolated and tested. Adoptive transfer experiments using T cell clones and the T cell deficient nude mouse model of chlamydial genital tract infection demonstrate that both $CD4^+$ and $CD8^+$ T cell clones are capable of resolving infection.[19,20] The protective T-helper-cell clone in those studies was of the Th1 phenotype, secreting interferon-$\gamma$, IL-2 and TNF-$\alpha$.[20] Recipient mice receiving this clone and infected with *Chlamydia* produced low levels of anti-chlamydial antibody, suggesting that protection might result from immune effector functions other than antibody. A $CD8^+$ T cell clone has also been shown to be capable of resolving infection in nude mice.[19] The ability of the $CD8^+$ T cell clone to resolve infection was not as pronounced as the $CD4^+$ clone (55% vs. 81% resolution, respectively), but nevertheless a positive effect was observed. A common feature of the protective $CD4^+$ and $CD8^+$ clones was production of interferon-$\gamma$ and TNF-$\alpha$, which has led to the suggestion that these cytokines play an importantt role in protective immunity.

Because of the obligate intracellular lifestyle of chlamydiae, cytotoxic T cells (CTL) are thought to contribute to protective immunity. Until recently, investigators were unable to demonstrate convincingly that CTLs functioned in the anti-chlamydial immune response.[25,35,36] However, recent studies have demonstrated that MHC class I restricted CTLs are capable of lysing *Chlamydia*-infected cells.[4,51] The function of chlamydial CTLs in vivo is not understood, but the adoptive transfer of a CTL line decreased the chlamydial burden in systemically infected mice and the effect was dependent upon interferon-$\gamma$.[51] Obviously, though, those studies need to be confirmed in a more relevant model of mucosal chlamydial infection. Thus, our understanding of the role of $CD8^+$ T cells in host immunity to chlamydial infection is incomplete, and additional studies are needed to determine more precisely the importance of these cells and the mechanism(s) by which they exert their effect in vivo.

### Recent Investigations Using Gene Knockout Mice to Study Immunity To *C. trachomatis* Genital Tract Infection

The following data come from our recent studies using gene knockout mice to delineate the roles of T cell subpopulations and antibody in immunity to chlamydial genital tract infection. We recognize that the murine model of chlamydial genital tract infection has limitations in regard to its applicability to human infection. However, the murine model does allow us to analyze many aspects of host immunity, and provides a basis from which to formulate studies to address immunity to human chlamydial infection.

Gene targeting, a method by which specific genes are altered in embryonic stem cells and subsequently passed through the germ line, has been used to generate mice that are devoid of cell surface expression of either MHC class I, MHC class II, or Ig molecules. MHC class I-deficient animals have been generated by inactivation of the gene for $\beta_2$-microglobulin,[24] which is required for the proper assembly and cell surface expression of the MHC class I molecule; as a result these mice are deficient in $CD8^+$ T cells. Mice that are devoid of cell surface expression of MHC class II molecules, derived by inactivation of the I-$A_\beta$ gene, are deficient in $CD4^+$ T cells.[16] B cell development is arrested at the pre-B cell stage in mice having a targeted disruption of the $\mu$M gene, and these mice lack cell surface IgD and IgM and fail to produce immunoglobulin (Ig).[23] In our recent studies we examined the capacity of MHC class I-, MHC class II-, or Ig-deficient mice to resolve *C. trachomatis* genital tract infection and to resist secondary infectious challenge. A very brief summary of our findings are presented in Table 1.

To delineate the possible roles of serum and secretory antibody, and MHC class I- and class II-restricted T cell responses in the development of protective immunity to chlamydial infection, we evaluated chlamydial genital tract infection in specific gene knockout mice.[28] Female mice were infected vaginally with *C. trachomatis* strain MoPn and infection was monitored by swabbing the vaginal vault and enumerating inclusion forming units on a HeLa cell monolayer. Control mice and mice deficient in either MHC class I molecules or Ig resolved primary chlamydial infection by about 4 weeks post-infection. Shedding of infectious chlamydiae was not significantly different at any time following infection. Conversely, mice deficient in MHC class II molecules failed to resolve infection, and remained culture positive and continued to shed large numbers of chlamydiae (> 100,000 IFUs), throughout the observation period (>70 days).

The humoral and cell-mediated anti-chlamydial immune responses were evaluated during the course of primary chlamydial infection. Control and MHC class I-deficient mice produced high titers of serum anti-chlamydial antibodies, consisting primarily of IgG2a, IgG2b, and IgA subclasses. Ig deficient mice did not produce detectable anti-chlamydial antibodies and MHC class II-deficient mice produced only low levels of IgG2b and IgG3 antibodies. Vaginal washes were analyzed for chlamydial-reactive antibody, and only control and MHC class I-deficient mice were found to be positive (anti-*Chlamydia* IgA). Control, MHC class I-deficient and Ig-deficient mice had comparable chlamydial-specific CMI responses (delayed type hypersensitivity responses, T cell proliferation) following primary infection, whereas MHC class II-deficient mice were negative. Collectively, those results suggested that MHC class II-restricted T cell responses were necessary to bring about the resolution of primary chlamydial genital tract infection, whereas infection resolved typically in the absence of antibody or MHC class I-restricted T cell responses.

Following the resolution of primary chlamydial genital tract infection control, MHC class I-deficient and Ig-deficient mice were rechallenged to assess the development of acquired immune protection. Control and MHC class I-deficient mice were resistant to reinfection. Although some mice were reinfectable (~30%) they shed fewer infectious chlamydiae (4 to 5 $\log_{10}$ lower than primary infection) and for a shorter period of time (7–10 days vs. 28–30 days for a primary infection). Conversely, all Ig-negative mice were susceptible to reinfection, but this secondary infection was characterized by the shedding of fewer chlamydiae and an infection of shortened duration.

Our results from studying chlamydial genital tract infection in gene knockout mice reveal that:

**TABLE 1. Summary of immune responses and infection outcome following genital tract infection of control and gene knockout mice[a]**

| | Mouse Strain | | | |
|---|---|---|---|---|
| | Control | MHC class I deficient | MHC class II deficient | B cell deficient |
| Resolve 1° infection | yes | yes | no | yes |
| Anti-*Chlamydia* antibodies | | | | |
| serum | yes | yes | no[b] | no |
| vaginal wash | yes | yes | no | no |
| Anti-chlamydial CMI | yes | yes | no | yes |
| Resistant to 2° Infectious challenge | yes | yes | NT[c] | no |

[a]Refer to the text for detailed explanation of these findings.
[b]Low levels of anti-*Chlamydia* IgG2b and IgG3 were detected.
[c]Not tested.

1) MHC class II-restricted T cell responses are absolutely necessary to resolve primary infection; 2) MHC class I-restricted responses are neither necessary to bring about the resolution of primary infection nor to resist secondary challenge. However, we can not exclude the possibility that those responses play an important role in immunity to chlamydial infection in the immunocompetent animal; 3) Cell-mediated immunity plays a dominant role in resolving established chlamydial infection; 4) The presence of local (vaginal) anti-*Chlamydia* antibody at the time of inoculation prevents colonization and subsequent infection.

### SUMMARY

*Chlamydia*-specific antibody and CMI responses play key roles in immunity to chlamydial genital tract infection. Local IgA antibodies appear to promote resistance to reinfection, whereas CMI responses do not protect against reinfection per se, but instead promote the resolution of established infection. Although the presence of antibody at the site of infection (genital tract) can prevent colonization/infection, sterilizing immunity through neutralizing antibody might be difficult to achieve. If these findings hold for human chlamydial infection, then the therapeutic value of a vaccine solely targeted to elicit neutralizing antibody might be debatable. Although such a vaccine would be ideal, it is unlikely that long-lived protective immunity could be achieved using a vaccine designed to elicit only neutralizing antibodies. Perhaps a better vaccine strategy might be to concentrate efforts on immunizations that stimulate protective cell-mediated immunity, or cell-mediated immunity and neutralizing antibody. Although significant advances have been achieved in describing protective immune responses to chlamydial genital tract infection, gaps still remain in our understanding of host immunity. The effector cells of CMI that resolve infection have neither been characterized nor their antigenic specificity determined. Furthermore, once protective antigens have been identified it must be determined if they can be administered in a manner that stimulates protective immunity in a naive host.

### REFERENCES

1. Alani MD, Darougar S, Burns DC, Thin RN, Dunn H: Isolation of *Chlamydia trachomatis* from the male urethra. Br J Vener Dis 53:88–92, 1977.
2. Barenfanger J, MacDonald AB: The role of immunoglobulin in the neutralization of trachoma infectivity. J Immunol 113:1607–1617, 1974.
3. Batteiger BE, Rank RG: Analysis of the humoral immune response to chlamydial genital infection in guinea pigs. Infect Immun 55:1767–1773, 1987.
4. Beatty PR, Stephens RS: CD8$^+$ T lymphocyte-mediated lysis of *Chlamydia*-infected L cells using an endogenous antigen pathway. J Immunol 153:4588–4595, 1994.
5. Brunham RC, Kuo C-C, Cles L, Holmes KK: Correlation of host immune response with quantitative recovery of *Chlamydia trachomatis* from the human endocervix. Infect Immun 39:1491–1494, 1983.
6. Brunham RC, Martin DH, Kuo C-C, Wang S-P, Stevens CE, Hubbard T, Holmes KK: Cellular immune response during uncomplicated genital infection with *Chlamydia trachomatis* in humans. Infect Immun 34:98–104, 1981.
7. Brunham RC, Peeling R, Maclean I, McDowell J, Persson K, Osser S: Postabortal *Chlamydial trachomatis* salpingitis: Correlating risk with antigen-specific serological responses and with neutralization. J Infect Dis 155:749–755, 1987.

8. Byrne GL, Grubbs B, Dickey TJ, Schachter J, Williams DM: Interferon in recovery from pneumoniae due to *Chlamydia trachomatis* in the mouse. J Infect Dis 156:993–996, 1987.
9. Cain TK, Rank RG: Local Th1-like responses are induced by intravaginal infection of mice with the mouse pneumonitis biovar of *Chlamydia trachomatis*. Infect Immun 63:1784–1789, 1995.
10. Cevenini R, Rumpianesi F, Donati M, Moroni A, Sambri V, La Placa M: Class specific immunoglobulin response to individual polypeptides of *Chlamydia trachomatis*, elementary bodies, and reticulate bodies in patients with chlamydial infection. J Clin Pathol 39:1313–1316, 1986.
11. Cevenini R, Rumpianesi F, Sambri V, La Placa M: Antigenic specificity of serological response in *Chlamydia trachomatis* urethritis detected by immunoblotting. J Clin Pathol 39:325–327, 1986.
12. Cevenini R, Sarov I, Rumpianesi F, Donati M, Melega C, Varotti C, La Placa M: Serum specific IgA antibody to *Chlamydia trachomatis* in patients with chlamydial infections detected by ELISA and an immunofluorescence test. J Clin Pathol 37:686–691, 1984.
13. Darougar S: The humoral immune response to chlamydial infection in humans. Rev Infect Dis 7:726–730, 1985.
14. Grayston JT, Wang S-P: New knowledge of chlamydiae and the diseases they cause. J Infect Dis 132:87–104, 1975.
15. Grayston JT, Wang S-P, Yeh L-J, Kuo C-C: Importance of reinfection in the pathogenesis of trachoma. Rev Infect Dis 7:717–725, 1985.
16. Grusby MJ, Johnson RS, Papaioannou VE, Glimcher LH: Depletion of $CD4^+$ T cells in major histocompatibility complex class II-deficient mice. Science 253:1417–1420, 1991.
17. Hallberg T, Wolner-Hanssen P, Mardh P-A: Pelvic inflammatory disease in patients infected with *Chlamydia trachomatis:* In vitro cell mediated immune response to chlamydial antigens. Genitourin Med 61:247–251, 1985.
18. Hanna L, Schmidt L, Sharp M, Stites DP, Jawetz E: Human cell-mediated immune responses to chlamydial antigens. Infect Immun 23:412–417, 1979.
19. Igietseme JU, Magee DM, Williams DM, Rank RG: Role for $CD8^+$ T cells in antichlamydial immunity defined by chlamydia-specific T-lymphocyte clones. Infect Immun 62:5195–5197, 1994.
20. Igietseme JU, Ramsey KH, Magee DM, Williams DM, Kincy TJ, Rank RG: Resolution of murine chlamydial genital infection by the adoptive transfer of a biovar-specific, Th1 lymphocyte clone. Regional Immunol 5:317–324, 1994.
21. Jawetz E, Rose L, Hanna L, Thygeson P: Experimental inclusion conjunctivitis in man. Measurements of infectivity and resistance. JAMA 194:620–632, 1965.
22. Katz BP, Batteiger BE, Jones RB: Effect of prior sexually transmitted disease on the isolation of *Chlamydia trachomatis*. Sex Transm Dis 14:160–164, 1987.
23. Kitamura D, Roes J, Kuhn R, Rajewsky K: A B cell-deficient mouse by targeted disruption of the membrane exon of the immunoglobulin $\mu$ chain gene. Nature 350: 423–426, 1991.
24. Koller BH, Smithies O: Inactivating the $\beta_2$-microglobulin locus in mouse embryonic stem cells by homologous recombination. Proc Natl Acad Sci USA 86:8932–8935, 1989.
25. Lammert JK: Cytotoxic cells induced after *Chlamydia psittaci* infection in mice. Infect Immun 35:1011–1017, 1982.
26. Landers DV, Erlich K, Sung M, Schachter J: Role of L3T4-Bearing T-Cell populations in experimental murine chlamydial salpingitis. Infect Immun 59:3774–3777, 1991.
27. Mardh PA: Chlamydial pelvic inflammatory disease. In: P. Reeve (ed.): Chlamydial Infections. Berlin: Springer-Verlag, pp. 45–55.
28. Morrison RP, Feilzer K, Tumas DB: Gene knockout mice establish a primary protective role for major histocompatibility complex class II-restricted responses in *Chlamydia trachomatis* genital tract infection. Infect Immun 63:4661–4668, 1995.
29. Murray ES, Charbonnet LT, MacDonald AB: Immunity to chlamydial infections of the eye. I. The role of circulatory and secretory antibodies in resistance to reinfection with guinea pig inclusion conjunctivitis. J Immunol 110:1518–1525, 1973.
30. Murray ES, Radcliffe FT: Immunologic studies in guinea pigs with guinea pig inclusion conjunctivitis (GP-IC) Bedsonia. Am J Ophthalmol 63:1263–1269, 1967.
31. Newhall V WJ, Batteiger VB, Jones RB: Analysis of the human serological response to proteins of *Chlamydia trachomatis*. Infect Immun 38:1181–1189, 1982.
32. Nichols RL, Murray ES, Nisson PE: Use of enteric vaccines in protection against chlamydial infections of the genital tract and the eye of guinea pigs. J Infect Dis 138:742–746, 1978.
33. Nichols RL, Oertley RE, Fraser CEO, MacDonald AB, McComb DE: Immunity to chlamydial infections of the eye. VI. Homologous neutralization of trachoma infectivity for the owl monkey conjunctivae by eye secretions from humans with trachoma. J Infect Dis 127:429–432, 1973.
34. Patton DL, Rank RG: Animal models for the study of pelvic inflammatory disease. In: Gallin JI, Fauci AS (eds.): Advances in host defense mechanisms, 8th ed. New York: Raven Press, Ltd., pp. 85–111.
35. Pavia CS, Schachter J: Failure to detect cell-mediated cytotoxicity against *Chlamaydia trachomatis*-infected cells. Infect Immun 39:1271–1274, 1983.
36. Qvigstad E, Hirschberg H: Lack of cell-mediated cytotoxicity towards *Chlamydia trachomatis* infected target cells in humans. Acta Pathol Microbiol Immunol Scand (C) 92:153–159, 1984.
37. Ramsey KH, Newhall, V WJ, Rank RG: Humoral immune response to chlamydial genital infection of mice with the agent of mouse pneumonitis. Infect Immun 57:2441–2446, 1989.
38. Ramsey KH, Rank RG: The role of T cell subpopulations in the resolution of chlamydial genital tract infection of mice. In: Bowie WR, Caldwell HD, Jones RP, Mardh P-A, Ridgway GL, Schachter J, Stamm WE, Ward ME (eds.), Proceedings of the Seventh International Symposium on Human Chlamydial Infections. Cambridge: Cambridge University Press, pp. 241–244.

39. Ramsey KH, and Rank RG: Resolution of chlamydial genital infection with antigen-specific T-lymphocyte lines. Infect Immun 59:925–931, 1991.
40. Ramsey KH, Soderberg LSF, Rank RG: Resolution of chlamydial genital infection in B-cell-deficient mice and immunity to reinfection. Infect Immun 56:1320–1325, 1988.
41. Rank RG, Barron AL: Humoral immune response in acquired immunity to chlamydial genital infection of female guinea pigs. Infect Immun 39:463–465, 1983.
42. Rank RG, Barron AL: Specific effect of estradiol on the genital mucosal antibody response in chlamydial ocular and genital infections. Infect Immun 55:2317–2319, 1987.
43. Rank RG, Batteiger BE, Soderberg LSF: Susceptibility to reinfection after a primary chlamydial genital infection. Infect Immun. 56:2243–2249, 1988.
44. Rank RG, Soderberg LSF, Barron AL: Chronic chlamydial genital infection in congenitally athymic nude mice. Infect Immun 48:847–849, 1985.
45. Rank RG, Soderberg LSF, Sanders MM, Batteiger BE: Role of cell-mediated immunity in the resolution of secondary chlamydial genital infection in guinea pigs infected with the agent of guinea pig inclusion conjunctivitis. Infect Immun 57:706–710, 1989.
46. Rank RG, White HJ, Barron AL: Humoral immunity in the resolution of genital infection in female guinea pigs infected with the agent of guinea pig inclusion conjunctivitis. Infect Immun 26:573–579, 1979.
47. Schachter J: Chlamydial infections (First of three parts). N Engl J Med 298:428–435, 1978.
48. Schachter J: Chlamydial infections (Second of three parts). N Engl J Med 298:490–495, 1978.
49. Schachter J: Chlamydial infections (Third of three parts). N Engl J Med 298:540–548, 1978.
50. Schachter J, Cles LD, Ray RM, Hesse FE: Is there immunity to chlamydial infections of the human genital tract? Sex Transm Dis 10:123–125, 1983.
51. Starnbach MN, Bevan MJ, Lampe MF: Protective cytotoxic T-lymphocytes are induced during murine infection with *Chlamydia trachomatis.* J Immunol 153:5183–5189, 1994.
52. Su H, Caldwell HD: $CD4^+$ T cells play a significant role in adoptive immunity to *Chlamydia trachomatis* infection of the mouse genital tract. Infect Immun 63:3302–3308, 1995.
53. Tuffrey M, Falder P, Taylor-Robinson D: Genital-tract infection and disease in nude and immunologically competent mice after inoculation of a human strain of *Chlamydia trachomatis.* Br J Exp Pathol 63:539–546, 1982.
54. Tuffrey M, Falder P, Taylor-Robinson D: Effect of *Chlamydia trachomatis* infection of the murine genital tract on adoptive transfer of congenic immune cells or specific antibody. Br J Exp Pathol 66:427–433, 1985.
55. Wagar EA, Schachter J, Bavoil P, Stephens RS: Differential human serologic response to two 60,000 molecular weight *Chlamydia trachomatis* antigens. J Infect Dis 162:922–927, 1990.
56. Wang S-P, Grayston JT, Alexander ER: Trachoma vaccine studies in monkeys. Am J Ophthalmol. 63:1615–1630, 1967.
57. Watson RR, Mull JD, MacDonald AB, Thompson III SE, Bear SE: Immunity to chlamydial infections of the eye. II. Studies of passively transferred serum antibody in resistance to infection with ginea pig inclusion conjunctivitis. Infect Immun 7:597–599, 1973.
58. Williams DM, Grubbs B, Schachter J: Primary murine *Chlamydia trachomatis* pneumonia in B-cell-deficient mice. Infect Immun 55:2387–2390, 1987.
59. Williams DM, Magee DM, Bonewald LF, Smith JG, Bleicker CA, Byrne GI, Schachter J: A role in vivo for tumor necrosis factor alpha in host defense against *Chlamydia trachomatis.* Infect Immun 58:1572–1576, 1990.
60. Williams DM, Schachter J, Grubbs B, Sumaya CV: The role of antibody in host defense against the agent of mouse pneumonitis. J Infect Dis 145:200–205, 1982.
61. Williams DM, Schachter J, Weiner MH, Grubbs B: Antibody in host defense against mouse pneumonitis agent (murine *Chlamydia trachomatis*). Infect Immun 45:674–678, 1984.
62. Woolridge RL, Grayston JT, Chang IH, Yang CY, Cheng KH: Long-term follow-up of the initial (1959–1960) trachoma vaccine field trial on Taiwan. Am J Ophthalmol 63:1650–1655, 1967.
63. Zhang Y-X, Stewart S, Joseph T, Taylor HR, Caldwell HD: Protective monoclonal antibodies recognize epitopes located on the major outer membrane protein of *Chlamydia trachomatis.* J Immunol 138:575–581, 1987.
